# In-hospital acute kidney injury and atrial fibrillation: incidence, risk factors, and outcome

**DOI:** 10.1080/0886022X.2021.1939049

**Published:** 2021-06-21

**Authors:** Guoqin Wang, Lijiao Yang, Nan Ye, Weijing Bian, Changsheng Ma, Dong Zhao, Jing Liu, Yongchen Hao, Na Yang, Hong Cheng

**Affiliations:** a Division of Nepphrology, Beijing Anzhen Hospital, Capital Medical University, Beijing, China; b Department of Cardiology, Beijing Anzhen Hospital, Capital Medical University, Beijing, China; c Department of Epidemiology, Beijing Anzhen Hospital, Capital Medical University, Beijing Institute of Heart, Lung and Blood Vessel Diseases, Beijing, China

**Keywords:** Acute kidney injury, atrial fibrillation, risk stratification, outcome

## Abstract

**Background:**

The incidence and the risk factors of in-hospitalized acute kidney injury (AKI) in patients hospitalized for atrial fibrillation (AF) were unclear.

**Methods:**

The Improving Care for Cardiovascular Disease in China-AF (CCC-AF) project is an ongoing registry and quality improvement project, with 240 hospitals recruited across China. We selected 4527 patients hospitalized for AF registered in the CCC-AF from January 2015 to January 2019. Patients were divided into the AKI and non-AKI groups according to the changes in serum creatinine levels during hospitalization.

**Results:**

Among the 4527 patients, the incidence of AKI was 8.0% (361/4527). Multivariate logistic analysis results indicated that the incidence of in-hospital AKI in patients with AF on admission was 2.6 times higher than that in patients with sinus rhythm (OR 2.60, 95% CI 1.77–3.81). Age (per 10-year increase, OR 1.22, 95% CI 1.07–1.38), atrial flutter/atrial tachycardia on admission (OR 2.16, 95% CI 1.12–4.15), diuretics therapy before admission (OR 1.48, 95% CI 1.07–2.04) and baseline hemoglobin (per 20 g/L decrease, OR 1.21, 95% CI 1.10–1.32) were independent risk factors for in-hospital AKI. β blockers therapy given before admission (OR 0.67, 95% CI 0.51–0.87) and non-warfarin therapy during hospitalization (OR 0.71, 95% CI 0.53–0.96) were associated with a decreased risk of in-hospital AKI. After adjustment for confounders, in-hospital AKI was associated with a 34% increase in risk of major adverse cardiovascular (OR 1.34, 95% CI 1.02–1.90, *p* = 0.023).

**Conclusions:**

Clinicians should pay attention to the monitoring and prevention of in-hospital AKI to improve the prognosis of patients with AF.

## Introduction

Atrial fibrillation (AF) is the most common heart rhythm disorder. The estimated number of individuals with AF globally in 2010 was 335 million [1]. There were significant variations in prevalence between regions, and developed countries had higher prevalence rates than developing countries [1]. In China, the age-standardized prevalence of AF in the general population was 0.65%, reaching 7.50% in >80-year-olds [2].

Acute kidney injury (AKI) is recognized as one of the most serious complications in hospitalized individuals. AKI can lead to increased mortality and treatment costs of hospitalized patients, so the prevention of AKI has great significance in clinical practice. In 2015, the in-hospital AKI reported detection rate in China was 2.3%, which was much lower than that reported in developed countries (7–18%). The possible reason is that doctors neglect in-hospital AKI [3]. At present, increasing attention has been paid to the occurrence of in-hospital AKI in patients with cardiovascular diseases such as acute coronary syndrome and acute heart failure, and the incidence of nosocomial AKI is gradually decreasing [4]. Due to the need for long-term anticoagulant therapy in patients with AF, studies on the occurrence of renal injury caused by warfarin and novel anticoagulant drugs have been reported [5]. However, there are very few studies on the incidence of in-hospital AKI in patients with AF, especially in China. Approximately 10–40% of patients with AF are hospitalized each year [6]. It has been reported that in-hospital AKI is associated with higher mortality in elderly patients with AF [7]. Therefore, the identification of risk factors for in-hospital AKI in patients with AF will improve the monitoring of high-risk groups by doctors, reduce the incidence of in-hospital AKI and thus improve the prognosis of patients with AF.

In this study, we investigated the incidence of in-hospital AKI in patients hospitalized for AF, observed the effects of in-hospital AKI on in-hospital outcomes and analyzed the risk factors for in-hospital AKI.

## Materials and methods

### Research design

Details of the design and methodology of the Improving Care for Cardiovascular Disease in China-AF (CCC-AF) project have been published [8]. In brief, it is a national, hospital-based quality improvement project with an ongoing database, aiming to increase adherence to AF guidelines in China and to improve patient outcomes. It was launched in 2014 as a collaborative initiative of the American Heart Association (AHA) and the Chinese Society of Cardiology (CSC). A total of 240 hospitals were recruited, representing the diversity of AF care in hospitals in China, including 150 tertiary hospitals in phase I and phase II and 82 secondary hospitals and eight tertiary hospitals in phase III (from July 2017) and phase IV (from November 2018). In each hospital, the first 10–20 hospitalized patients with AF are enrolled in a consecutive manner. Clinical data were collected *via* a web-based data collection platform (Oracle Clinical Remote Data Capture, Oracle Corporation). Trained data extractors entered the data elements extracted from medical charts. Eligible patients were consecutively reported to the CCC-AF database for each month before the middle of the following month. Approximately 5% of reported cases were randomly selected and compared with the original medical records. An audit by a third party was performed to ensure that cases were reported consecutively rather than selectively. From January 2015 to January 2019, the clinical data of 48542 in-patients with AF were collected.

This study protocol was approved by the Ethics Committee of Beijing Anzhen Hospital, Capital Medical University. A waiver for the need to obtain informed consent was obtained, given the nature of the study. This research has been registered at https://clinicaltrials.gov (NCT02306616).

### Research population

We included patients hospitalized for AF. All patients were diagnosed with AF by electrocardiograph (ECG) results, which were recorded by 12-lead ECG, 24-h Holter ECG or other cardiac rhythm monitors (such as pacemaker, ICD). An arrhythmia that has the ECG characteristics of AF and lasts sufficiently long for a 12-lead ECG to be recorded or is otherwise documented to last for at least 30 s, should be considered to be an AF episode. In addition to the duration requirements listed above, the diagnosis of AF requires an ECG or rhythm strip demonstrating: (1) ‘absolutely’ irregular R-R intervals (in the absence of complete atrioventricular [AV] block); (2) no distinct P waves on the surface ECG; and (3) an atrial cycle length (when visible) that is usually less than 200 ms [9]. The patients with two serum creatinine record in the hospital were collected in this study. Patients with AF secondary to reversible conditions (e.g., untreated thyroid disease and pulmonary embolism) were excluded from the study. Patients were divided into the AKI group and the non-AKI group according to the changes in serum creatinine levels during hospitalization.

### Research variables

The serum creatinine (SCr) level at admission was the baseline SCr level. The estimated glomerular filtration rate (eGFR) of patients was calculated by the Chronic Kidney Disease Epidemiology Collaboration (CKD-EPI) equation [10]. The diagnosis of AKI depended on the changes in serum creatinine levels during hospitalization. The definition of AKI was based on the Kidney Disease: Improving Global Outcomes (KDIGO) criteria: an increase in the SCr level ≥50% or ≥26.5 μmol/L, which is known or presumed to have occurred within seven days [11]. Patients who had nephrectomy or kidney transplantation were excluded. According to the KDIGO criteria, AKI stage 1 was defined as an increase in SCr ≥1.5 times the baseline value or an increase in SCr ≥ 26.5 μmol/L; stage 2 was defined as an increase in SCr 2.0–2.9 times the baseline value; and stage 3 was defined as an increase in SCr 3.0 times the baseline value or ≥4.0 mg/dL (353.6 μmol/L). Urine output measurements were not available in the data set and were not included in the definition of AKI used in this analysis. Previous medical history was provided by the patient or previous medical records. The diagnosis of hypertension was based on the previous history of hypertension and/or a measurement of systolic blood pressure ≥140 mmHg and/or diastolic blood pressure ≥90 mmHg after admission or discharge diagnosis. AF subtype was adopted the AF classification system that was presented in the 2014 AHA/ACC/HRS Guideline for the management of patients with atrial fibrillation [9].

### Research outcomes

The primary outcome of this study was in-hospital AKI. The second outcomes included major adverse cardiovascular and cerebrovascular events (MACCEs) and bleeding events during hospitalization. MACCEs included cardiovascular death, heart failure, cardiac arrest, cardiogenic shock and ischemic stroke/transient ischemic attack (TIA). The bleeding events included cerebral bleeding, gastrointestinal bleeding and mucocutaneous bleeding before 2017. After 2017, according to the international society on thrombosis and hemostasis (Isth) criteria, CCC-AF included the information of patients with severe bleeding and mild bleeding. The used criteria for major bleeding were the International Society on Thrombosis and Hemostasis (ISTH) criteria (Supplemental Method).

### Statistical analysis

Continuous variables are presented as the means and standard deviations or medians and interquartile ranges when the distribution and variance met the conditions. Categorical variables are presented as percentages. The comparisons between groups of continuous variables were performed by one-way ANOVA or the Mann–Whitney U-test (Kruskal–Wallis), and the chi-square test was used to compare the categorical variables. We analyzed relevant covariates that might be associated with in-hospital AKI with multivariate logistic regression. The covariates included sex (male vs. female), age (per 10 years increase), history of heart failure, antiarrhythmic drug before admission, β blockers before admission, diuretics before admission, cardiac rhythm on admission, baseline eGFR, baseline hemoglobin (HGB) (per 20 g/L decrease), CHA_2_DS_2-_VASc score, HAS-BLED score, percutaneous coronary intervention (PCI) during hospitalization, the use of nonwarfarin during hospitalization. The association between in-hospital AKI and MACCEs was also analyzed with a logistic regression model. The adjustment factors included sex (male vs. female), age (per 10-year increase), a history of heart failure, a history of coronary heart disease, a history of cerebrovascular disease, AF subtype, cardiac rhythm on admission, baseline eGFR and warfarin treatment during hospitalization. A multivariate logistic regression model was used to determine the association between in-hospital AKI and bleeding events with adjustment for sex (male vs. female), age (per 10-year increase), history of bleeding, AF subtype, cardiac rhythm on admission, baseline eGFR, use of anticoagulation therapy before admission, Warfarin treatment during hospitalization, CHA2DS2-VASC score and HAS-BLED score. For variables with missing values, the sequential regression multiple imputation method implemented by IVEware version 0.2 (Survey Research Center, University of Michigan, Ann Arbor, MI, USA) was used to impute the missing values. All P values were 2-tailed, and *p* < 0.05 was considered statistically significant. Statistical analyses were performed using SPSS 23.0 (SPSS Inc., Chicago, IL).

## Results

### Detection rate of AKI

**Figure 1.**
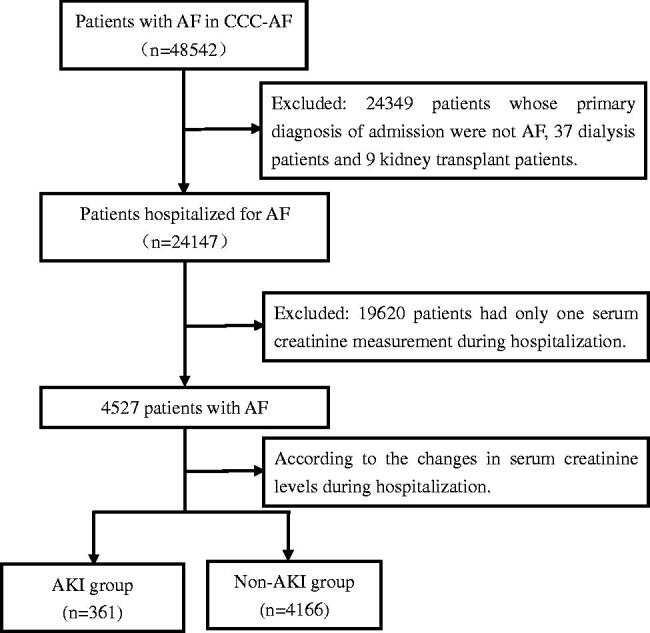
Flow chart of the patients enrolled in this study.

**Figure 2.**
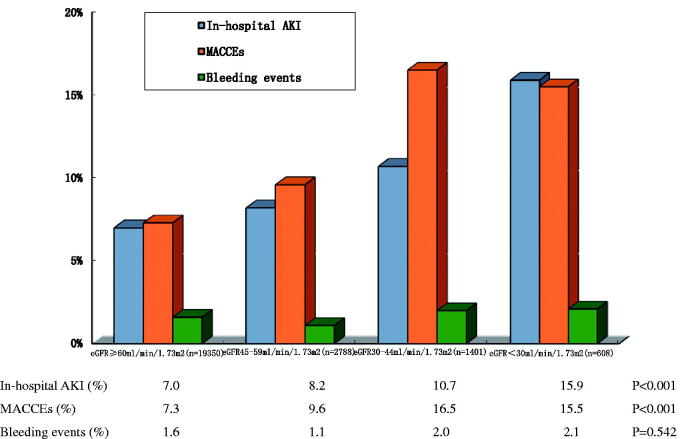
The rates of in-hospital AKI, MACCEs and bleeding events observed according to baseline eGFR of patients with AF. MACCEs include cardiovascular death, heart failure, cardiac arrest, cardiogenic shock and ischemic stroke/TIA. Bleeding events include cerebral bleeding, gastrointestinal bleeding and mucocutaneous bleeding.

From January 2015 to January 2019, a total of 48,542 patients with AF admitted to the hospital were included in the CCC-AF. There were 24147 patients hospitalized for AF. A total of 19 620 of the 24 147 patients had only one SCr measurement during hospitalization and were excluded. Dialysis patients and kidney transplant patients were also excluded. A total of 4527 patients (4527/24 147, 18.7%) had their SCr levels measured twice, of whom 361 patients had AKI. The detection rate of AKI was 8.0% (361/5427), Figure 1. Among them, AKI 1 accounted for 84.8% (306/361), AKI 2 accounted for 11.4% (41/361) and AKI 3 accounted for 3.9% (14/361). In addition, according to the following order: baseline eGFR ≥ 60 mL/min/1.73 m^2^, 45–59 mL/min/1.73 m^2^, 30-44 mL/min/1.73 m^2^ and < 30 mL/min/1.73 m^2^, the detection rates of in-hospital AKI significantly increased, 7.0%, 8.2%, 10.7% and 15.9% (*p* < 0.001), Figure 2.

### Characteristics of AKI patients

The baseline characteristics of patients with or without AKI are shown in Table 1. Compared with the non-AKI group, the patients in the AKI group were older and had more heart failure and kidney disease. The percentages of AKI patients receiving diuretics before admission were significantly higher than those of non-AKI patients. The proportion of patients using pre-admission antiarrhythmic drugs and anticoagulant drugs were very low in the AKI group and non-AKI group, and antiarrhythmic drugs (7.2% vs. 11.9%), β-blockers (23.8% vs. 31.2%) and nonwarfarin anticoagulants (2.8% vs. 6.6%) were used relatively less frequently in the AKI group. Patients in the AKI group had lower baseline eGFRs and baseline HGB levels than those in the non-AKI group. Compared with patients in non-AKI group, CHA_2_DS_2-_VASc score (*p* < 0.001) and HAS-BLED score (*p* = 0.003) were significantly greater in patients in AKI group. There were significant differences in cardiac rhythm on admission between AKI group and non-AKI group (*p* < 0.001, respectively).

**Table 1.** Baseline characteristics of AF patients with or without AKI.

Characteristics	AKI group	Non-AKI group	*p* Value
	*n* = 361	*n* = 4166	
Male sex (%)	182 (50.4)	2187 (52.5)	0.448
Age (y)	72.20 ± 11.45	68.38 ± 11.77	<0.001
Age category (%)			<0.001
<65 y	96 (26.6)	1491 (35.8)	
65～75 y	91 (25.2)	1369 (32.9)	
>75 y	174 (48.2)	1306 (31.3)	
Admission path (%)			0.245
Emergency department	56 (15.5)	554 (13.3)	
Clinic department	292 (80.9)	3503 (84.1)	
Other	13 (3.6%)	109 (2.6)	
Comorbidities (%)			
Smoking	65 (18.0)	806 (19.3)	0.535
Hypertension	204 (56.5%)	2302 (55.5%)	0.646
Diabetes mellitus	72 (19.9)	734 (17.6)	0.268
Kidney disease	25 (6.9)	112 (2.7)	<0.001
Coronary heart disease	58 (16.1)	696 (16.7)	0.754
Heart failure	42 (11.6)	328 (7.9)	0.012
Cerebrovascular disease	50 (13.9)	624 (15.0)	0.564
Previous bleeding	5 (1.4)	75 (1.8)	0.566
AF subtype			0.008
First detected AF	66 (18.3)	764 (18.3)	
Persistent AF	124 (34.4)	1372 (32.9)	
Permanent/long standing persistent AF	11 (3)	100 (2.4)	
Paroxysmal AF	137 (38.0)	1802 (43.3)	
Presenting Rhythm on admission			<0.001
AF	310 (85.9)	3048 (73.2)	
Sinus rhythm	35 (9.7)	915 (22.0)	
Atrial flutter/Atrial tachycardia	15 (4.2)	168 (4.0)	
Paced	1 (0.3)	35 (0.8)	
Treatment at baseline (%)			
Antiarrhythmic drugs	26 (7.2)	497 (11.9)	0.007
β blockers	86 (23.8)	1301 (31.2)	0.003
Aspirin	64 (17.7)	745 (17.9)	0.941
ACEI/ARB	85 (23.5)	860 (20.6)	0.193
Anticoagulant drugs	61 (16.9)	964 (23.1)	0.007
Warfarin	53 (14.7)	3422 (14.4)	0.874
Nonwarfarin	10 (2.8)	275 (6.6)	0.004
Diuretic	57 (15.8)	440 (10.6)	0.002
Statins	51 (14.1)	646 (15.5)	0.486
Laboratory values at admission			
Serum creatinine (μmol/L)	103.33 ± 77.03	93.94 ± 60.31	0.006
eGFR (ml/min/1.73 m^2^)	67.74 ± 27.04	72.00 ± 22.92	<0.001
＜60 ml/min/1.73 m^2^	141 (39.1)	1235 (29.6)	<0.001
Potassium (mmol/L)	4.00 ± 0.59	4.05 ± 0.54	0.128
HGB (g/L)	123.99 ± 23.99	131.24 ± 24.59	<0.001
LVEF (%)	56.46 ± 12.38	58.51 ± 11.40	0.003
CHA_2_DS_2-_VASc score	3.27 ± 1.86	2.88 ± 1.73	<0.001
HAS-BLED score	1.57 ± 0.88	1.43 ± 0.88	0.003

eGFR: estimated glomerular filtration rate; HGB: hemoglobin; LVEF: left ventricular ejection fraction.

With regard to nosocomial treatment, we noted a low rate of surgical treatment in both groups but a significantly lower rate of radiofrequency ablation (13.0% vs. 26.9%) in the AKI group than in the non-AKI group. In the AKI group, the proportion of patients receiving anticoagulant therapy after admission was significantly higher than that before admission; however, the proportions of patients in the AKI group receiving anticoagulant therapy and nonwarfarin anticoagulant therapy were still lower than those in the non-AKI group (Table 2). The length of hospitalization in the AKI group was significantly longer than that in the non-AKI group (10 (7, 14) days vs. 9 (7, 13) days, *p* = 0.034).

**Table 2.** Treatment and outcome of AF patients with and without AKI during hospitalization.

Treatment	AKI group	Non-AKI group	*p* Value
	*n* = 361	*n* = 4166	
**Treatment**			
Radiofrequency ablation (%)	47 (13.0)	1119 (26.9)	<0.001
Cardioversion (%)	4 (1.1)	111 (2.7)	0.071
PCI (%)	20 (5.5)	232 (5.6)	0.982
Pacemaker implantation (%)	11 (3.0)	183 (4.4)	0.226
Anticoagulant drugs	201 (55.7)	2843 (68.2)	<0.001
Warfarin (%)	140 (38.8)	1779 (42.7)	0.148
Nonwarfarin (%)	57 (15.8)	1013 (24.3)	<0.001
Aspirin	125 (34.6)	1195 (28.7)	0.017
β blockers	215 (59.6)	2317 (55.6)	0.148
**Outcome**			
MACCEs (%)	45 (12.5)	358 (8.6)	0.013
Heart failure (%)	40 (11.1)	301 (7.2)	0.008
Mortality (%)	3 (0.8)	3 (0.1)	<0.001
Cardiac arrest (%)	2 (0.6)	7 (0.2)	0.114
Cardiogenic shock (%)	1 (0.3)	5 (0.1)	0.432
Ischemic stroke/TIA (%)	3 (0.8)	63 (1.5)	0.300
Bleeding events (%)	9 (2.5)	61 (1.5)	0.129
Severe bleeding*	0	8 (0.2)	<0.001

PCI: percutaneous coronary intervention; TIA: transient ischemic attack.

MACCEs include cardiovascular death, heart failure, cardiac arrest, cardiogenic shock and ischemic stroke/TIA. The bleeding events include cerebral bleeding, gastrointestinal bleeding and mucocutaneous bleeding.

*Data collected after 2017.

### Risk factors for in-hospital AKI

Multivariate logistic regression analysis was used to screen for risk factors for in-hospital AKI in patients with AF, Table 3. The results indicated that increased age (per 10-year increase), the use of diuretics before admission (OR 1.48, 95% CI 1.07–2.04) and baseline hemoglobin (per 20 g/L decrease) were independent risk factors for in-hospital AKI after adjustment. Compared with patients with sinus rhythm on admission, patients with AF and patients with atrial flutter/atrial tachycardia on admission increased in-hospital AKI risk by 2.08-fold (*p* < 0.001) and 2.16-fold (*p* = 0.021). In addition, β blockers therapy given before admission (OR 0.67, 95% CI 0.51–0.87) and non-warfarin therapy during hospitalization (OR 0.71, 95% CI 0.53–0.96) were associated with a decreased risk of in-hospital AKI.

**Table 3.** Logistic regression analysis of the risk factors for in-hospital AKI in patients with AF.

	Unadjusted		Adjusted	
	OR (95% CI)	*p* Value	OR (95% CI)	*p* Value
Age (per 10-year increase)	1.33	<0.001	1.22	0.003
	(1.21, 1.46)		(1.07, 1.38)	
Sex (male vs female）	0.92	0.448	1.16	0.258
	(0.74, 1.14)		(0.89, 1.49)	
History of heart failure	1.54	0.013	1.25	0.238
	(1.10, 2.17)		(0.87, 1.80)	
Antiarrhythmic drug before admission	0.57	0.008	0.79	0.278
	(0.38, 0.86)		(0.52, 1.21)	
β blockers before admission	0.69	0.004	0.67	0.003
	(0.54, 0.88)		(0.51, 0.87)	
diuretics before admission	1.59	0.002	1.48	0.018
	(1.18, 2.14)		(1.07, 2.04)	
Cardiac rhythm on admission				
Sinus rhythm	Ref.		Ref.	
AF	2.66	<0.001	2.60	<0.001
	(1.86, 3.80)		(1.77, 3.81)	
Atrial flutter/Atrial tachycardia	2.33	0.008	2.16	0.021
	(1.25, 4.37)		(1.12, 4.15)	
Paced	0.75	0.777	0.63	0.614
	(0.10, 5.61)		(0.08, 4.60)	
AF subtype				
First detected AF	Ref.		Ref.	
Persistent AF	0.91	0.496	0.81	0.165
	(0.68, 1.21)		(0.60, 1.09)	
Permanent /long-standing persistent AF	1.10	0.772	0.96	0.898
	(0.570, 2.13)		(0.49, 1.88)	
Paroxysmal AF	0.76	0.056	0.99	0.986
	(0.58, 1.01)		(0.74, 1.34)	
Baseline eGFR	0.93	<0.001	1.00	0.477
	(0.92, 0.94)		(0.99, 1.01)	
HGB (per 20 g/L decrease)	1.24	<0.001	1.21	<0.001
	(1.15, 1.34)		(1.10, 1.32)	
CHA_2_DS_2-_VASc score	1.13	<0.001	1.03	0.502
	(1.07, 1.21)		(0.94, 1.13)	
HAS-BLED score	1.20	0.003	1.03	0.747
	(1.06, 1.36)		(0.87, 1.21)	
PCI during hospitalization	1.00	0.982	1.11	0.663
	(0.62, 1.59)		(0.69, 1.80)	
Nonwarfarin during hospitalization	0.58	<0.001	0.71	0.024
	(0.44, 0.78)		(0.53, 0.96)	

eGFR: estimated glomerular filtration rate; PCI: percutaneous coronary intervention; HGB: hemoglobin.

ORs was adjusted for sex (male vs female), age (per 10 years increase), history of heart failure, antiarrhythmic drug before admission, β blockers before admission, diuretics before admission, cardiac rhythm on admission, AF subtype, baseline eGFR, baseline HGB (per 20 g/L decrease), CHA_2_DS_2-_VASc score, HAS-BLED score, PCI during hospitalization, Nonwarfarin during hospitalization.

### Association of in-hospital AKI with MACCEs and bleeding events

The incidence rates of MACCEs in the AKI group were significantly higher than those in the non-AKI group, and no difference was observed in the incidence of bleeding events between groups, as shown in Table 2 and Supplemental Table 1. Furthermore, according to the following order: baseline eGFR ≥ 60 mL/min/1.73 m^2^, 45–59 mL/min/1.73 m^2^, 30–44 mL/min/1.73 m^2^ and < 30 mL/min/1.73 m^2^, we found that the detection rates of MACCEs significantly increased as 7.3%, 9.6%, 16.5% and 15.5% (*p* < 0.001). However, there was no such change trend in in-hospital bleeding events (*p* = 0.542) (Figure 2).

**Figure 3.**
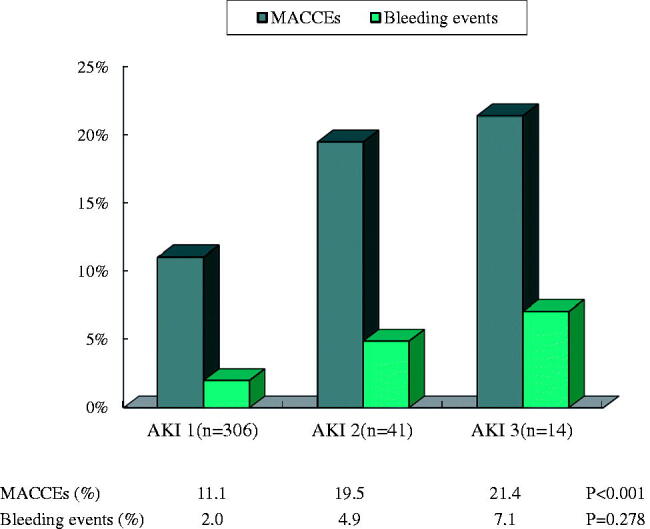
Incidence of MACCEs and bleeding events in patients with different AKI stages. MACCEs include cardiovascular death, heart failure, cardiac arrest, cardiogenic shock and ischemic stroke/TIA. Bleeding events include cerebral bleeding, gastrointestinal bleeding and mucocutaneous bleeding.

In patients with AKI, the incidence of MACCEs increased significantly with the increasing severity of AKI (*p* < 0.001). Although bleeding events increased with the increasing severity of AKI, there was no significant difference among the three stages (2.0%, 4.9% and 7.1%, *p* = 0.278) (Figure 3).

We analyzed the associations between in-hospital AKI and MACCEs and bleeding events. After adjustment, we found that in-hospital AKI was an independent risk factor for MACCEs (OR = 1.34, 95% CI 1.02 to 1.90, *p* = 0.023) (Table 4, Supplemental Table 2), but not for bleeding events (OR = 1.76, 95% CI 0.86 to 3.62, *p* = 0.124) (Supplemental Table 2).

**Table 4.** Logistic regression analysis for MACCEs and bleeding events in AF patients with and without AKI.

	Unadjusted		Adjusted	
	OR (95% CI)	*p* Value	OR (95% CI)	*p* Value
MACCEs#				
AKI	1.52	0.014	1.34	0.023
	(1.09, 2.11)		(1.02, 1.90)	
Bleeding events*				
AKI	1.72	0.133	1.76	0.124
	(0.85, 3.49)		(0.86, 3.62)	

MACCEs include cardiovascular death, heart failure, cardiac arrest, cardiogenic shock and ischemic stroke/TIA. Bleeding events include cerebral bleeding, gastrointestinal bleeding and mucocutaneous bleeding.

#Adjusted by sex (male vs. female), age (per 10-year increase), history of heart failure, history of coronary heart disease, history of cerebrovascular disease, AF subtype, cardiac rhythm on admission, baseline eGFR, Warfarin treatment during hospitalization.

*Adjusted by sex (male vs. female), age (per 10-year increase), history of bleeding, AF subtype, cardiac rhythm on admission, baseline eGFR, use of anticoagulation therapy before admission, and Warfarin treatment during hospitalization, CHA2DS2-VASC score, HAS-BLED score.

## Discussion

Among hospitalized patients with cardiovascular diseases, ACS, acute and chronic heart failure and cardiac surgery patients are more likely to develop AKI. However, the incidence of AKI in hospitalized patients with AF has not been reported in China thus far. In this study, we observed the occurrence of AKI in patients hospitalized for AF in 240 tertiary hospitals in China. Among the 24 147 hospitalized patients with AF, 361 of the 4527 patients who had SCr levels tested more than twice developed AKI, and the incidence of AKI was 8.0%. In 2015, Yang [3] reported that the detection rate of AKI in Chinese hospitals was only 2.03%, which is lower than the 7% to 18% reported in developed countries [12]. The author believes that due to doctors' insufficient attention to AKI, only 25.3% of the patients admitted to the hospital had repeated SCr assays, which is much lower than the figure reported in developed countries (63.2–67.6%) [4,12]. Similarly, in CCC-AF, only 18.7% (4527/24147) of the patients hospitalized for AF received two SCr tests. However, the incidence of in-hospital AKI in this study was close to the data reported by other studies [12]. AF is a common cardiovascular disease in the elderly population. The number of AF patients aged ≥35 years is 5.26 million according to the 2010 Chinese Census. The number of patients with new-onset AF is increasing every year, especially among elderly patients with hypertension, diabetes, kidney disease and other complications [13]. AF can increase all-cause mortality by 1.46 times, cardiogenic mortality by 2.03 times, and the risk of all-cause mortality by 1.66 times in CKD patients [14]. In-hospital AKI is an important risk factor for in-hospital mortality, especially in patients with cardiovascular disease [4,15]. Therefore, in-hospital AKI in patients with AF should be considered. In patients with a high risk of AKI, SCr levels should be closely monitored to detect AKI as early as possible [16]. In our study, we investigated the incidence of in-hospital AKI in patients with AF in China and analyzed the risk factors for in-hospital AKI to improve the prognosis of patients with AF.

This study analyzed the risk factors for AKI in hospitalized patients with AF, and the results showed that the traditional risk factors for AKI, such as age, were also independent risk factors for in-hospital AKI in patients with AF. Patients with AF have a 64% higher risk of a decrease in the eGFR due to concomitant hypertension and diabetes and warfarin use [14]. Individuals with kidney dysfunction are more likely to develop hypertension and have worse control of their blood pressure, which leads to left ventricular hypertrophy, poor ventricular compliance, and eventually atrial stretch and fibrosis, which are established predictors of AF [17]. In addition, CKD can lead to pathological activation of the intrarenal renin–angiotensin–aldosterone system. An upregulated renin–angiotensin–aldosterone system causes atrial fibrosis and electrical remodeling, increasing the risk of AF [18]. Therefore, AF and CKD are closely related diseases [14,17]. In our study, compared with patients with sinus rhythm on admission, patients with AF and patients with atrial flutter/atrial tachycardia on admission significantly increased in-hospital AKI risk, and the use of β-blockers before admission may decrease the risk of in-hospital AKI. So, heart rate control is beneficial for prevention of in-hospital AKI in AF patients. In addition, although logistic analysis showed that the baseline eGFR decreased was not association with the risk of in-hospital AKI. However, the incidence of in-hospital AKI rises with decreasing baseline eGFR. Therefore, AF patients with CKD should be monitored for changes in renal function.

Previous studies have confirmed that the prognosis of AF patients with CKD is significantly worse than that of AF patients without CKD [19]. A retrospective cohort study by Nelson *et al.* observed 55 962 patients with CKD, and the results showed that survival after incident AF decreased progressively as CKD stage increased, and the one-year mortality rate for patients with CKD stages 3 to 5 with AF was 35.6% [19]. However, there have been few studies on the association between in-hospital AKI and adverse hospital prognosis in patients with AF [7,20]. Veleiro reported that 804 patients with AF aged >75 years were hospitalized for any cause, of whom 119 (14.8%) developed in-hospital AKI, and AKI was associated with the in-hospital mortality of patients (OR 2.4, 95% CI 1.03–5.53) [7]. Chan *et al.* retrospectively observed 3497677 AF hospitalizations, 3751 (0.11%) of which were complicated by AKI requiring dialysis (AKI-D), which was associated with mortality and adverse discharge [20]. In our study, the mortality of AF patients with AKI was significantly higher than that of AF patients without AKI, but the mortality rate of AF patients with AKI was only 0.8%, which was significantly lower than that of the general population reported by other studies (10.8–12.4%) [3]. The reason for the low mortality rate may be that the severity of AKI in our study was relatively mild, and only 3.9% of patients with stage-3 AKI, which is much lower than that reported by other studies (18.5%–28.6%) [3]. We observed associations between in-hospital AKI and MACCEs and bleeding events, and the results were similar to those of other studies [3,4]. In-hospital AKI was an independent risk factor for MACCEs in hospitalized patients with AF, but not for bleeding events. Even after adjustment for baseline eGFR, in-hospital AKI increased the risk of MACCEs by 1.34 times. It is suggested that more attention should be paid to the associations between AKI and MACCEs in patients with AF during hospitalization, but the effect of in-hospital AKI on long-term prognosis needs to be studied further.

Our study also had some limitations. First, this study was cross sectional based on CCC-AF database and retrospectively identified patients with in-hospital AKI. Some variables are not collected in CCC-AF. In additional, the temporal relationship between in-hospital AKI and clinical outcomes could not be assessed. We will add these variables in the continuous improvement of the database and investigate the results further in additional prospective studies. Second, this study was a cross-sectional study of patients with AF during hospitalization, without observation and analysis of the long-term outcomes in patients with in-hospital AKI. Long-term follow-up of these patients is needed in future studies to observe the recovery of kidney function in patients with AKI.

## Conclusion

This is the largest sample size multi-center study on in-hospital AKI in patients with AF in China, and it involved hospitals from multiple regions. The results showed that the detection rate of in-hospital AKI in Chinese patients with AF is 8.0%. The risk factors for in-hospital AKI were age (per 10-year increase), AF on admission, atrial flutter/atrial tachycardia on admission, the use of diuretics before admission and baseline hemoglobin (per 20 g/L decrease). β blockers therapy given before admission and non-warfarin therapy during hospitalization decreased the risk of in-hospital AKI. In-hospital AKI was an independent risk factor for MACCEs in patients with AF. It is important to identify groups at high risk for in-hospital AKI and reduce the occurrence of in-hospital AKI in patients with AF. Our study will help doctors identify patients at high risk for in-hospital AKI, prompting them to closely monitor renal function and reduce the incidence of in-hospital AKI in patients with AF.

## Supplementary Material

Supplemental Material
