# Supplementary material for: In-hospital acute kidney injury and atrial fibrillation: incidence, risk factors, and outcome
Source: Ren Fail. 2021 Jun 21;43(1):949–57. doi: 10.1080/0886022X.2021.1939049 (PMC8218696; doi:10.1080/0886022X.2021.1939049)
Supplement: Supplemental Material [file IRNF_A_1939049_SM6348.pdf]

Supplemental Data

Supplemental Materials and Methods

AF subtype

we have adopted the AF classification system that was presented in the 2014 AHA/ACC/HRS Guideline for the Management of Patients with Atrial Fibrillation. ①Paroxysmal AF is defined as AF that terminates spontaneously or with intervention within 7 days of onset; ②persistent AF is defined as continuous AF that is sustained beyond 7 days; ③long-standing persistent AF is defined as continuous AF of greater than 12 months' duration. ④The term permanent AF is defined as AF in which the presence of the AF is accepted by the patient and physician, and no further attempts will be made to either restore or maintain sinus rhythm.

Major bleeding

The most commonly used criteria for anticoagulation-related major bleeding is the International Society on Thrombosis and Haemostasis (ISTH) criteria: ① Fatal bleeding, and/or, ② Symptomatic bleeding in a critical area or organ, such as intracranial, intraspinal, intraocular, retroperitoneal, intraarticular or pericardial, or intramuscular with compartment syndrome, and/or, ③ Bleeding causing a fall in hemoglobin level of 2 g/dL or more, or leading to transfusion of two or more units of whole blood or red cells.

Supplementary Table 1. Logistic regression analysis of the risk factors for MACCE

	Unadjusted		Adjusted	
	OR (95% CI)	*P*-value	OR (95% CI)	*P*-value
Cardiac rhythm on admission				
Sinus rhythm	Ref.		Ref.	
AF	3.86 (2.61, 5.71)	<0.001	2.76 (1.82, 4.19)	<0.001
Paced	3.82 (2.08, 6.99)	<0.001	3.35 （1.80, 6.26）	<0.001
Atrial flutter/Atrial tachycardia	4.12 （1.36, 12.43）	0.012	2.20 （0.69, 7.07）	0.184
AF subtype				
First detected AF	Ref.		Ref.	
Persistent AF	2.00 (1.51, 2.66)	<0.001	1.58 （1.18, 2.12）	0.020
Permanent /long standing persistent AF	0.86 0.39, 1.93)	0.718	0.59 （0.26, 1.34）	0.207
Paroxysmal AF	0.87 (0.64, 1.18)	0.360	1.15 (0.84, 1.58)	0.388
AKI	1.52 (1.09, 2.11)	0.014	1.34 (1.02, 1.90)	0.023
history of coronary heart disease	1.50 (1.17, 1.92)	0.001	1.19 (0.91, 1.56)	0.206
history of cerebrovascular disease	1.42 (1.09, 1.85)	0.009	1.18 (0.90, 1.56)	0.238
Age (per 10-year increase)	1.20 (1.09, 1.31)	<0.001	1.03 (0.93, 1.15)	0.558
history of heart failure	5.96 (4.64, 7.66)	<0.001	4.70 (3.61, 6.12)	<0.001
Sex (male vs female）	0.86 (0.70, 1.06)	0.147	0.91 (0.74, 1.14)	0.418
Baseline eGFR	0.99 (0.98, 0.99)	<0.001	0.99 (0.99, 1.00)	<0.001
Warfarin treatment during hospitalization	1.29 (1.05, 1.59)	0.015	1.298 (1.04, 1.61)	0.019

Adjusted by sex (male vs female), age (per 10-year increase), history of heart failure, history of coronary heart disease, history of cerebrovascular disease, AF subtype, cardiac rhythm on admission, baseline eGFR, Warfarin treatment during hospitalization

Supplementary Table 2. Logistic regression analysis of the risk factors for bleeding events

	Unadjusted		Adjusted	
	OR (95% CI)	*P*-value	OR (95% CI)	*P*-value
Cardiac rhythm on admission				
Sinus rhythm	Ref.		Ref.	
AF	1.81 (0.89, 3.66)	0.101	1.27 (0.59, 2.74)	0.547
Paced	0	0.998	0	0.998
Atrial flutter/Atrial tachycardia	2.34 （0.71, 7.67）	0.162	1.71 （0.50,5.80）	0.389
AF subtype				
First detected AF	Ref.		Ref.	
Persistent AF	1.45 (0.79, 2.69)	0.235	1.24 (0.65, 2.37)	0.510
Permanent /long standing persistent AF	0	0.996	0	0.996
Paroxysmal AF	0.74 (0.38, 1.43)	0.370	0.74 （0.37, 1.48）	0.399
AKI	1.72 (0.85, 3.49)	0.133	1.76 (0.86, 3.62)	0.124
history of bleeding	5.55 (2.33, 13.23)	<0.001	5.41 (2.04, 14.33)	0.001
Age (per 10-year increase)	1.08 (0.88, 1.31)	0.468	1.14 (0.86, 1.52)	0.355
Anticoagulation therapy before admission	1.47 （0.88, 2.47）	0.141	1.32 (0.76, 2.28)	0.326
Warfarin treatment during hospitalization	2.40 (0.87, 6.61)	0.090	1.41 (0.86, 2.30)	0.174
Sex (male vs female）	0.64 (0.40, 1. 03)	0.068	0.56 (0.63, 0.96)	0.036
Baseline eGFR	0.99 (0.98, 1.00)	0.159	0.99 (0.98, 1.01)	0.299
CHA_2DS_2-VASc score	0.99 (0.86, 1.13)	0.857	0.85 (0.69, 1.06)	0.156
HAS-BLED score	1.06 (0.81, 1.39)	0.665	1.00 (0.70, 1.44)	0.999

Adjusted by sex (male vs female), age (per 10-year increase), history of bleeding, AF subtype, cardiac rhythm on admission, baseline eGFR, use of anticoagulation therapy before admission, and Warfarin treatment during hospitalization, CHA2DS2-VASC score, HAS-BLED score
